# On Acquired Syphilis

**Published:** 1871-04

**Authors:** William Wilson


### Selections.

### ON ACQUIRED SYPHILIS.

BY WILLIAM WILSON, M.D., M.Ch., Q.U.I.

I have to submit to the profession the following notes of an extremely interesting case of acquired syphilis, which came under my notice while acting as "*locum tenens*" for the assistant-surgeon in the County Down Infirmary.

It will be seen from the notes of the case that it favors the view which Ricord entertained—*viz.*, that a wet-nurse may contaminate the infant to which she gives suck through the medium of her milk. Acton denies the possibility of this; but the following case is strongly in favor of Ricord's views. Another vexed question is resuscitated in this case—*i. e.*, whether or no a syphilitic child can affect a healthy nurse. Both Ricord and Acton deny the possibility of this; whereas Hunter and Lawrence cite cases in which it has occurred. Rollet, in his report, "Recherches Cliniq et Experiment sur la Syphilis," as quoted by Lancereaux in his "Treatise on Syphilis" (vol. ii, p. 246 et seq.), maintains the view of saliva acting as an infecting medium. The case related by Rollet is so interesting that I am sure I will be excused for quoting it here: "A young woman of irreproachable morals contracted syphilis, the first manifestation of which was a chancre on the lips. After questioning the patient in the presence of her mother and husband, Rollet came to the conclusion that the disease had been communicated by the cook. The latter, who had been ill for eight or ten months, had the isthmus of the throat occupied by confluent mucus patches, and the young lady was in the habit of tasting all dishes prepared by the servant with the same spoon, and immediately after her."

In the case which I am about to quote, there is the most striking similarity, in the means whereby the syphilis was transmitted, to the above case of Rollet's.

*Case.*—Biddy M'Veigh, æt. 33, married, by occupation a housewife, living at Kilkeel, was admitted into Downpatrick Infirmary on December 8th, 1870, and placed under the care of Dr. Maconchy for secondary syphilis. Also, at the same time, her four children—*viz.*, Maryanne, æt. 11; Eliza, æt. 7; William, æt. 2; Biddy, æt. 1—were admitted for the same affection. The mother stated, on admission, that she had been laboring for some time under sore throat, but that she had been taking medicine for it, and that now her throat was almost quite well, and that she only wished admission to look after her children. On examination, the tonsils of the mother were found to bear the marks of superficial ulceration, but there were no signs of inflammation, and she did not complain of any pain. The children were all found to be laboring under ulcerated sore throat, the tonsilitic ulcers having the bi-symmetrical character and gouged out appearance peculiar to specific sores. In addition, all the children had those serpiginous patches round the margin of the anus which are known as condylomata. None of the family had any other affection, with the exception of the youngest child—Biddy, æt. 1—who had small superficial ulcers about the angles of the mouth, roseola over the lower two-thirds of the nose, and larygitic symptoms; also, aphthous-looking spots on the tongue. The mother complained of nothing, nor did she present any symptoms of the disease except the condition of the throat before referred to. The eldest child, Maryanne, had ulcerated sore throat of a more virulent character, and condylomata larger and more irritable than the rest of the family. With these exceptions, there was no difference of any moment in the symptoms between the rest of the members of the family. The youngest child, in consequence of its present constitutional disorder, looked very debilitated; the others were strong, tolerably healthy-looking children, full of animal spirits.

On inquiry, the mother stated that the youngest child was the first to be affected. That it was, on the day of its birth, a fine healthy child; and that on that day her sister-in-law—a married woman—gave the child *the breast* on two separate occasions. She (the mother) being told by a neighbor that her sister-in-law was laboring under the "*bad disorder*"—having been smitten by her husband—took the child from the sister-in-law, and never again allowed her to suckle the child on any other occasion. From that time her child remained quite healthy, and sucked well for nearly a month; but when the child was close upon being four weeks old, she observed it becoming very cross and irritable, and she began to find great difficulty in inducing it to take the breast. About this time also—four weeks after birth—she observed spots coming out over the child's body of a dark red color, and about the size of a pea, which "*never came to a head*"—*i. e.*, never became purulent or serous. On seeing the eruption she got alarmed, and consulted a medical man, who gave her a *salve* and medicine; persevered in the use of the salve and medicine, and the spots disappeared in three months. About two months after she first saw the eruption, she observed the sores about the child's anus. Her medical man being consulted about these sores, pronounced them syphilitic, and ordered her to apply the "black wash" to keep the parts clean, and to give the child spoon diet, as the amount of nourishment it—of itself—was able to derive from the breast was inadequate for its sustenance. When the infant was two months old, she (the mother) observed two large flat-looking dark red spots on her breast—one on each side of the nipple of the breast she was accustomed to apply the child to—and shortly after she took a very bad sore throat, and used to spit out "*matter*" from it which had a bad taste and smell. It was nearly nine months before her throat was well, although during the whole of that time she had been under medical treatment. The states that she did not suffer from anything except the sore throat and the two spots on the breast.

The eldest daughter was accustomed to spoon-feed the youngest child, putting the spoonful of pap through her own mouth, in order to test whether or not it was too hot, etc., etc., before introducing it into the mouth of the child. When the whole spoonful was not swallowed by the child, she ate herself what it had left, before replenishing the spoon. Eliza and William, the other two children, were accustomed to receive spoonfulls of pap while the infant was being fed; and they came in generally for what was left on the spoon, after being passed through the child's mouth.

About five months after she first commenced to feed the child—as nearly as the mother can remember—Maryanne, the eldest daughter, complained of sore throat, and shortly afterwards showed her mother the condylomata round the margin of the anus. About three months after the eldest child first complained, the other two children—Eliza and William—also became affected with sore throat and condylomata. None of the children, with the exception of the youngest—who had roseola of the nose and small flat reddish "spots" over the body—had had any skin affection whatever; nor did the three eldest complain of anything except the throat affection and the condylomata.

Biddy M'Veigh (the mother), on being interrogated, stated that she had two children living at home with their father—one a boy, nine years old, and the other a girl, æt. 5. Neither of these children have, up to the present time, shown any symptoms of the disease. The mother states that the boy—who was very "*old fashioned*"—always refused to partake of the pap with which the infant was fed; he considered himself quite man, as he was accustomed to work in the fields with his father. She does not know how the other children escaped the affection. She thinks that the latter did not get any of the infant's food, but is not altogether clear on this latter point.

At the time the sister-in-law gave suck to the child, the mother saw her breast, and there were, in her opinion, neither "sores," "spots," nor anything else the matter with it; it was, in short, in her opinion, a healthy enough looking breast. She never heard from any one that there was anything wrong with her sister-in-law's breast. On being questioned, Biddy M'Veigh asserted, in the most positive manner, that her husband never had venereal disease; that he was, in fact, a "*very good living man.*" The husband also supported, with the most solemn asservations, the wife's statement as to his never having had venereal disease.

Biddy M'Veigh is a short, muscular, slightly swarthy woman, and pretty healthy-looking. She is intelligent, and her statements bear the impress of truth. At present she has been three months pregnant.

The whole family improved steadily under Dr. Maconchy's mercurial treatment, and at the present time (Jan. 5th, 1871) they are so far recovered as to be almost in a condition to leave the Infirmary.

On perusal of the above case, it will be noted that the youngest child became contaminated by a person who, as far as we can learn, had no cracks about the nipple, or excoriations of any other part of her breast, although laboring under constitutional syphilis at the time she gave suck to the child; and (2) That the child communicated syphilis to the mother, who had no abrasion on any part of her breast. That syphilis must have been communicated to the child through the medium of the milk, and the child infected the mother through the medium of the saliva. It will also be noted, that the eldest daughter—who had her mouth more constantly in contact with the spoon by which the child was fed—had more severe symptoms, and an earlier manifestation of the action of the syphilitic virus, than any of the other children; and, lastly, it will be noted, that the virus manifested its presence in all the members of the family, not by a primary sore, but by secondary symptoms.—*Journal of Cutaneous Medicine.*

